# Radiology practice in sub-Saharan Africa during the COVID-19 outbreak: points to consider

**DOI:** 10.11604/pamj.2020.37.28.23081

**Published:** 2020-09-07

**Authors:** Timothy Musila Mutala, Callen Kwamboka Onyambu, Angeline Anyona Aywak

**Affiliations:** 1 Department of Diagnostic Imaging and Radiation Medicine, School of Medicine, College of Health Sciences, University of Nairobi, Nairobi, Kenya

**Keywords:** COVID-19, radiology practice, sub-Saharan Africa

## Abstract

COVID-19 is a rapidly growing pandemic that has grown from a few cases in Wuhan, China to millions of infections and hundreds of thousands of deaths worldwide within a few months. Sub-Saharan Africa is not spared. Radiology has a key role to play in the diagnosis and management of COVID-19 as literature from Wuhan and Italy demonstrates. We therefore share some critical knowledge and practice areas for radiological suspicion and diagnosis. In addition, emphasis on how guarding against healthcare acquired infections (HAIs) by applying “red” and “green” principle is addressed. Given that pandemics such as COVID-19 can worsen the strain on the scantily available radiological resources in this region, we share some practical points that can be applied to manage these precious resources also needed for other essential services. We have noted that radiology does not feature in many main COVID-19 guidelines, regionally and internationally. This paper therefore suggests areas of collaboration for radiology with other clinical and management teams. We note from our local experience that radiology can play a role in COVID-19 surveillance.

## Perspectives

**Epidemiology:** COVID-19 was first described in Wuhan city, province of Hubei, China in December 2019 with subsequent genetic sequencing that gave definition of the novel coronavirus known as 2019 n-CoV or SARS-CoV-2 [1]. Within a short period of time it has spread across the world leading it to being declared a pandemic by Word Health Organization (WHO) on 11 March 2020. On 20 April 2020, the numbers stood at 2,411,553 and 165,338 for total number of cases and deaths respectively [2]. Though a seemingly late entrant in COVID-19 reports, Africa is also witnessing an upsurge with total cases almost tripling from 8,701 to 26,899 in a span of less than three weeks [3,4].

**Role of radiology in COVID-19:** radiology plays a significant role in management of COVID-19 patients, especially chest CT and chest X-ray [4-6]. Reports have also emerged on the utility of point of care ultrasound in management of this group of patients [7]. There are certain chest radiological features being attributed to COVID-19 from a sizeable database. These have been grouped into four categories namely: typical, indeterminate, atypical and negative for pneumonia emphasizing on the likelihood of COVID-19 infection according to the Radiological Society of North America (RSNA) [8]. A more or less similar approach for reporting in suspicion of COVID-19 lung disease is also emerging under CO-RADS, a standardized structured reporting tool developed by the Dutch Radiological Society. Radiologists in sub-Saharan Africa must be fully cognizant of the radiological features of COVID-19 by referring to the aforementioned literature and the few cases they have from imaging patients with COVID-19 disease in their local setting. These include ground glass opacification, mainly peripheral, basal and bilateral as well as multi-focal consolidations. These have been presented in major radiological and multidisciplinary forums and as such many radiologists are well versed with the imaging patterns of the disease. We highly encourage frequent refreshers at individual and larger group levels so as not to lose track of diagnosing complications in suspected cases or even pick clinically missed ones.

Indications for imaging in COVID-19 need to be clear to the clinicians and radiology teams to optimize benefits and risks. Imaging is not a screening tool for suspected COVID-19 and RT-PCR still remains the gold standard diagnostic method [7,9]. Again, not all diagnosed COVID-19 cases will require imaging and the strongest case for doing it is in setting and assessment of severe ARDS [5,9]. Utility of CT scan of the chest in probable cases as per the WHO case definition has been tried in some settings [10]. This consists of negative RT-PCR but highly suspicious symptomatology and history of contact and in some cases positive CT findings have preceded positive RT-PCR test. However, all the cases in the series finally turned positive at a later test and also a negative CT scan in such patients cannot constitute COVID-19 free status. From our country´s experience a few of the COVID-19 positive patients for whose imaging was indicated had findings not different from the pattern widely published.

**“Red” and “green” zones principle (minimize healthcare acquired infections):** diagnosis of COVID-19 starts at the public health and primary care realms with clear case definition as developed by WHO [11]. Patients are generally referred by clinicians for radiological examination. This means that imaging has to be clearly defined within national or regional COVID-19 handling management protocols and/or guidelines. Unfortunately, many national and international guidelines do not succinctly include radiology. Radiology can be either a problem solver or multiplier in COVID-19 management depending on how it is handled. The positive contributory factors are as highlighted in preceding section. The negative side is that general healthcare acquired infections (HAIs) have been associated with radiology units in some situations [12]. General preparedness in tackling HAIs through radiology varies across different countries within sub-Saharan Africa [13]. For that reason, national, regional and international infection prevention and control (IPC) guidelines should be made with input of radiology representatives. At the same time, in the main hospital setting, it is imperative that the radiology managers and their staff get actively involved in the development of their institutional standard operating protocols (SOPs) on handling COVID-19 cases. That way they will “ring fence” their departments from being conduits of HAIs to their staff and other patients within the unit.

**Figure 1:**
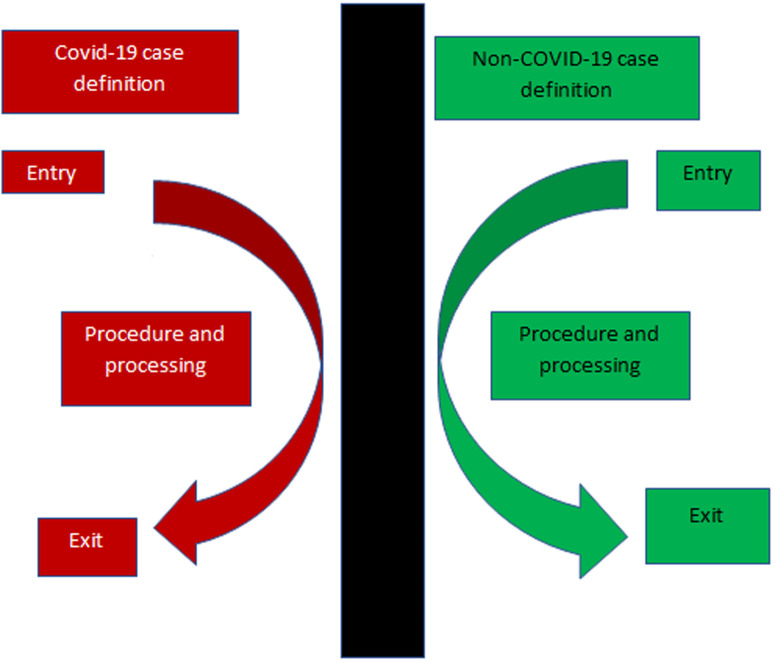
simplified demonstration of how thorough separation of COVID-19 and non-COVID-19 case definition patients must be handled in a designated facility

Patient traffic flow and handling in radiology during the COVID-19 era must be guided by the principle of “red” and “green” zone principles. This is generally described in a publication by Zhang *et al*. that captured the practice in Wuhan, China during the peak period of the outbreak there [14]. This is summarized in Figure 1. The definition of the zoning starts at the health facility´s main entrance where screening and triaging is thorough to isolate confirmed or suspected cases at the earliest opportunity. “Red zone” operations demand designation of facilities including imaging for the COVID-19 diagnosed or suspected patients. A call for national planners and institutional managers is to make sure that every isolation facility has designated imaging units, preferably mobile ones stationed within it. Radiological staff working within the “red” zone must be covered with adequate PPE as prescribed by authoritative health organizations [15]. We have noted that supply of PPEs continues to be a worldwide challenge, sub-Saharan Africa having its fair share of the same. Therefore, it is prudent that designated imaging facilities be the ones that handle COVID-19 patients to save on this scarce resource. This goes hand in hand with a primary triage that must be as watertight as possible to protect non-COVID-19 units (“green zones”) being inadvertently exposed. Enhanced IPC protocols for imaging in COVID-19 have been published including a video by Dr Pradeen Srinivasan of Fortis Hospital, Bangalore, India on their COVID-19 patient radiography procedure [16]. This has been adopted in our teaching facility, Kenyatta National Hospital and we highly recommend it. Another good reference for the in-depth detailing of the COVID-19 patient handling in the radiology department is also provided by Mossa-Basha *et al*. from their personal experience at University of Washington [17]. In short, “red” zone operations must be embraced by policy makers, health institution managers, radiology managers, radiologists and radiology technologists.

“Green” zone operations must also be emphasized so that no patient is denied essential diagnostic and interventional imaging services. Of course, services will operate under general public health measures of hygiene, social distancing and wearing of face masks. Spending the shortest time within the imaging facility should be a major goal of the managers and the entire team. Patient preparation procedures that can be done before arriving to the department should be encouraged. Examples include fluid intake before pelvic ultrasound and oral contrast for abdominal CT. Further, patients should be encouraged to come with their own drinking cups for such procedures. Also, it will be prudent to have patients not wait for the written reports within the facility as a way of managing queues. Facility managers and their staff must be fully versed with the disinfection protocols guided by equipment manufacturers. At least three vendors, namely GE Healthcare, Philips and Siemens Healthineers have published their explicit cleaning guides in their websites. The authors do not endorse any vendor products or recommendations and equipment owners are advised to check with their local representatives for clear instructions. With these IPC practice it is also important to note that decontamination of CT scan machines has been documented to result in a downtime running into hours [18]. This can lead to throughput issues for regular workflow if imaging is performed in non-designed facilities for COVID-19 cases.

**Challenges in radiology as part of the bigger healthcare system:** radiological equipment and imaging centers are a scarce resource for the sub-Saharan Africa population. It is our estimation that there are about 70 CT scans in Kenya serving a population of about 47 million people, giving a ratio of 1.49 units per million of the population [19,20]. Ngoya *et al*. report that there are 0.08 and 1.7 CT scan units per million for Tanzania and South Africa, respectively [21]. These are the confines within which imaging resources for COVID-19 will be integrated to. Hence more care in ensuring all the triage protocols are right. That sub-Saharan Africa is in dire need of radiologic human resource is not a new phenomenon [22,23]. From our experience as a training center of radiologists from several countries within sub-Saharan Africa since 1977, we also have firsthand experience on the matter. Our department has successfully trained about 200 radiologists who serve in Kenya, Tanzania, Malawi, Zambia, Zimbabwe, Botswana and Namibia among other countries. The radiologist to population ratio in sub-Saharan Africa is not flattering either. For example, Nigeria the most populous country in the region has 1: 566,000 and Kenya 1: 389,255 [24,25]. The ratio of other radiology workers, radiographers and physicists stands at 1: 63,845 in Kenya [26]. These are rare gems that must be handled with the greatest care during the COVID-19 pandemic. In tandem with international practice, exposed persons to COVID-19 in our country usually undergo mandatory quarantine for 14 days. This can have great impact on essential imaging services even for the “green” zone. Human resource managers should consider implementing non-contact rotational shifts for their staff to mitigate entire team grounding in the event of exposure. Another area to invest in human resource is through education, lack of which has already been cited as a possible cause of mortality from COVID-19 among health workers [27]. COVID-19 IPC education is available from many sources including the WHO website and many radiological society regular webinars. We encourage radiology to be given slots in local COVID-19 multidisciplinary webinars as has been the case in our country.

**Figure 2:**
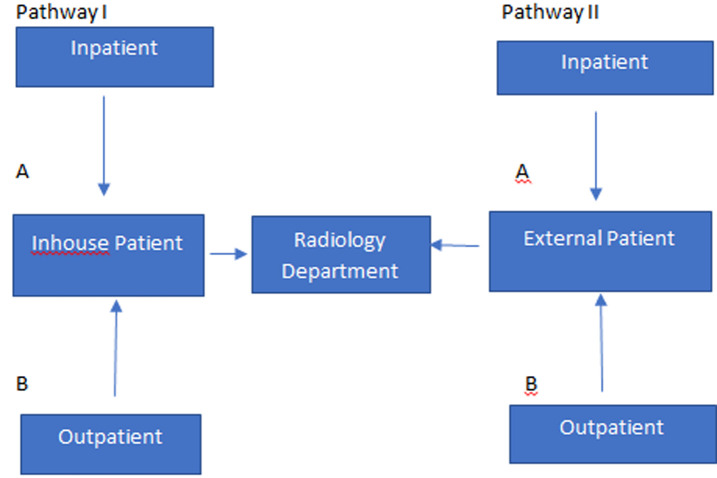
radiology department patient catchment scheme that can have implications on adherence of COVID-19 case handling SOPs. Pathways IA and IB should have the lowest risk in that regard, while pathway IIB would have the highest and IIA in between. Radiology managers need to incorporate referrals falling under II to their inhouse triage SOPs before proceeding with the examination

We envisage a challenge in sub-Saharan Africa arising from the ownership of the imaging facility. This is determined to a great extent on whether the department is part of a health institution like a hospital, mainly drawing its patients from within or a standalone facility which draws its patients from varied sources. In the former scenario, SOPs may be easily dovetailed within the system while in the latter this may not be realized. This is demonstrated in Figure 2 in which the inhouse referral is described as pathway I and external one pathway II. Further, we designate inpatients in both pathways as group “A” and outpatients as group “B”. In addition, private imaging facilities, especially the standalone ones can easily be overlooked in support. They can also lag behind in catching up with the necessary linkages for the fight against COVID-19. Thus, local authorities, professional societies and regulatory bodies must purposefully look for and support them so as to break any possible weak link.

**Radiology integration in COVID-19 activities:** radiological units operate in linkages with the clinical departments, peers, community, national and local authorities and other stakeholders. Collaborations must therefore be encouraged with all other stakeholders. Collaboration with regulatory bodies will play a key role in enforcement, where such is required. Professional societies must be very active in advocacy for best practice and at the same time in advising the policy makers in adjusting some approaches as far as COVID-19 response management is concerned. The most important collaboration is between the radiology and other clinical teams or referring chains that they interact with on daily operation activities.

**Radiological research and epidemiological surveillance in COVID-19:** COVID-19 is a field of research that will generate new knowledge to inform policy formulation and also help us become better radiology health workers in our service delivery. Some areas that will require research include the individual follow-up of the recovered patients and emerging imaging methods like point of care ultrasound (POCUS). The proportion of incidental diagnosis through radiological pathway in non-suspected cases will be of interest. Not to forget the impact of the pandemic on radiological resources and operations will need to be quantified and qualified at the same time. Research is best done through institutional and organizational collaborations and this is a good time for that. Funders can strongly consider radiological research for COVID-19 a priority given that many respiratory patients end up in imaging and also the radiology unit is a convergence zone for many other patients. In the same breath, utmost personal, patient and other players protection must be put into consideration when planning and implementing research related to COVID-19 as it can be highly contagious. All in all, it will be for the benefit of current and future generations that effective COVID-19 research is implemented in all spheres of diagnosis and care thus availability of PPEs for such a venture should be considered an investment. Epidemiological linkages will be key in informing radiology health workers in emerging cluster trends. At the same time radiology can be a good source for epidemiological teams to tap knowledge as far as suspicious imaging findings are reported. In fact, back in Wuhan it is reported through mainstream media that Dr Zhang Jixian raised the alarm of the COVID-19 outbreak by describing a pattern of CT chest abnormalities within a cluster of family members [28]. Considerations can be made by the surveillance teams to have tools that can capture this information developed in conjunction with and shared between radiology practitioners and them. We could not trace a radiological surveillance tool but our faculty is looking forward to developing one in partnership with the Ministry of Health, Kenya.

## Conclusion

Four key areas will ensure that radiology serves its best purpose during the COVID-19 outbreak in sub-Saharan Africa. These are good radiological knowledge of COVID-19 manifestations, “red and green” patient handling principle, prudent resource utilization and integration of radiology in epidemiological surveillance.
